# Supplementary material for: Giant anisotropic thermal expansion actuated by thermodynamically assisted reorientation of imidazoliums in a single crystal
Source: Nat Commun. 2019 Oct 22;10:4805. doi: 10.1038/s41467-019-12833-y (PMC6805950; doi:10.1038/s41467-019-12833-y)
Supplement: Supplementary file 1 — Supplementary Information [file 41467_2019_12833_MOESM1_ESM.docx]

**Supplementary Information for:**

**Giant anisotropic thermal expansion actuated by thermodynamically assisted reorientation of imidazoliums in a single crystal**

Yao et al.


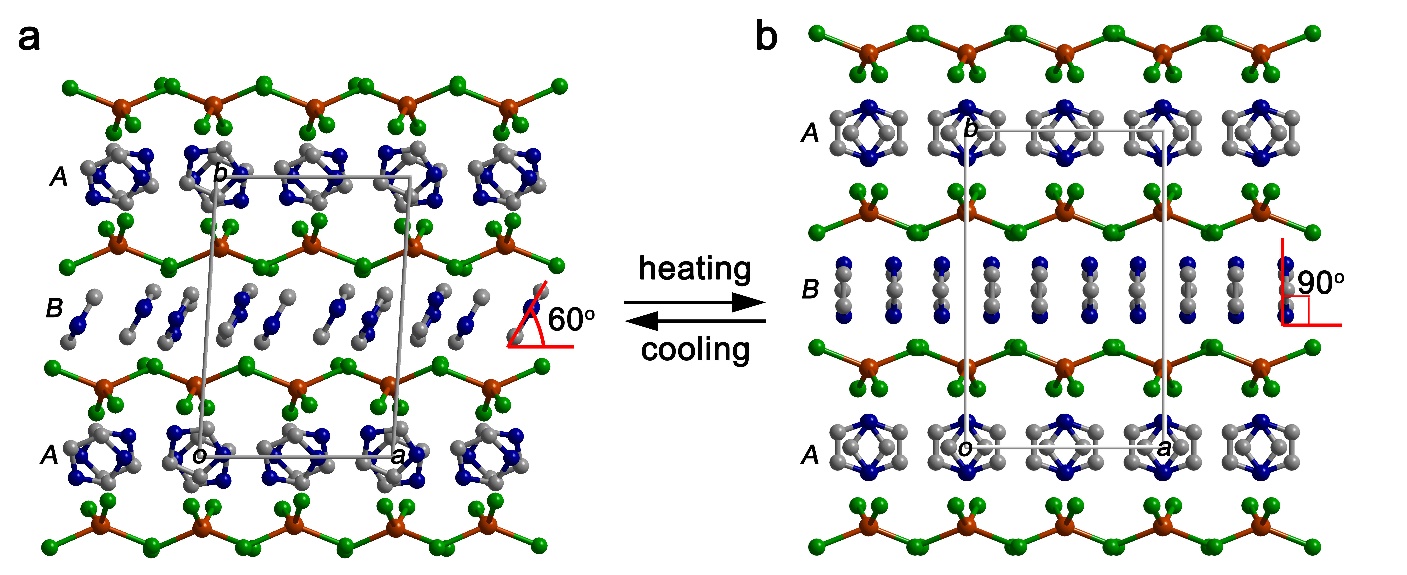


Supplementary Fig. 1 Crystal structures of 1 at 123 K (a) and 393 K (b). Molecular packing is viewed along the crystallographic c direction. Upon heating, the dihedral angle (θ) between the molecular plane of Himd^+^ in the layer B and crystallographic (010) face gradually increases and finally transforms to 90° in the HTP. Cu, brown; Cl, green; N, blue; C, grey; H, white-gray.


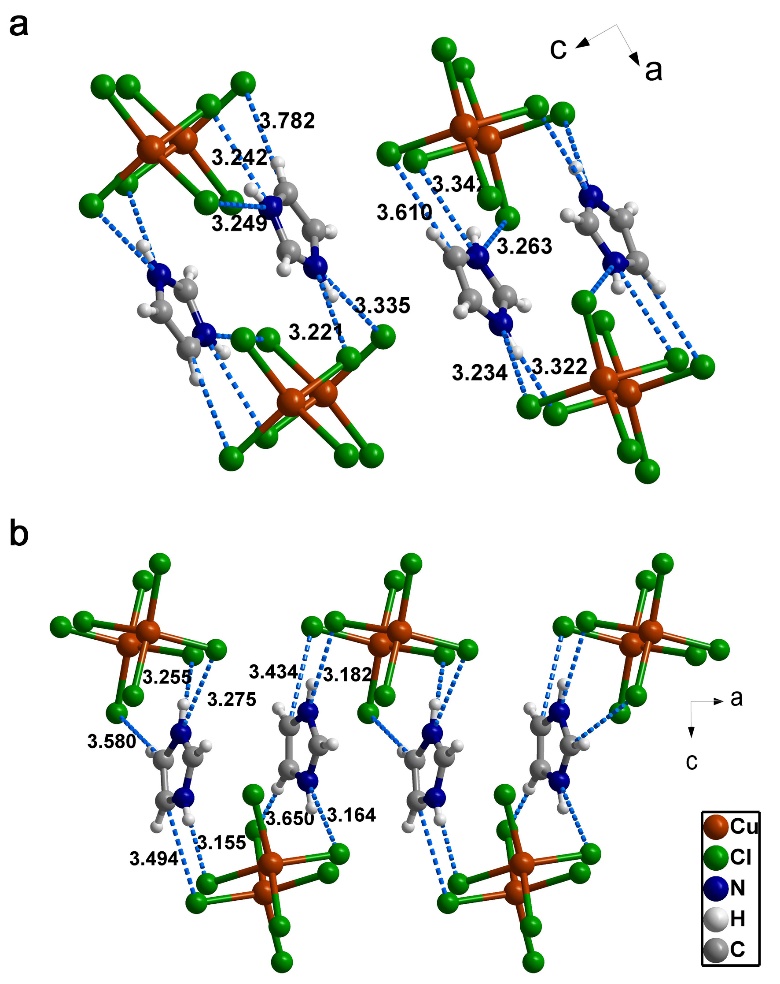


Supplementary Fig. 2 The hydrogen bond interactions between the Himd^+^ cations and CuCl_4_^2−^ dianions at 123 K. a, the hydrogen bond interactions of Himd^+^ cations in the A layers. Two Himd^+^ cations in A layer are surrounded by four CuCl_4_^2−^ anions, producing a rhomboid grid-like structure. b, the hydrogen bond interactions of Himd^+^ cations in the B layers. The Himd^+^ cations in layer B are connected in a zig-zag chain by CuCl_4_^2−^ anions along the crystallographic a direction. Cu, brown; Cl, green; N, blue; C, grey; H, white-gray.


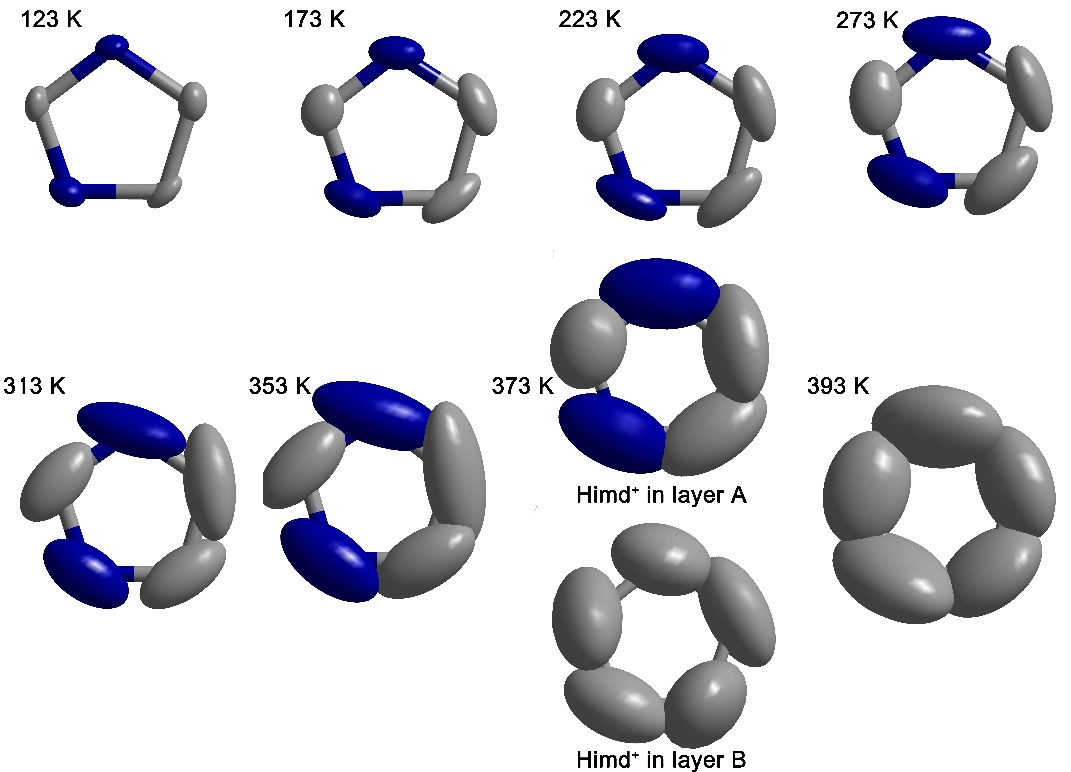


Supplementary Fig. 3 The thermal vibrations of cation at different temperatures. The intensity of the thermal vibration increases significantly as the temperature is increased. At 373 K, the Himd^+^ cations in layer A behave distinct in-plane wobbling, while those in layer B can rotate freely in the molecular plane according to the symmetry requirement of space group *P*2_1_/*c*. Upon further heating, the Himd^+^ cations in both the layers A and B become rotated. Notably, every non-hydrogen atoms in the rotated cations were refined with 60% C and 40% N. The thermal ellipsoids are drawn at a 50% probability level, and the hydrogen atoms are omitted for clarity. N, blue; C, grey.


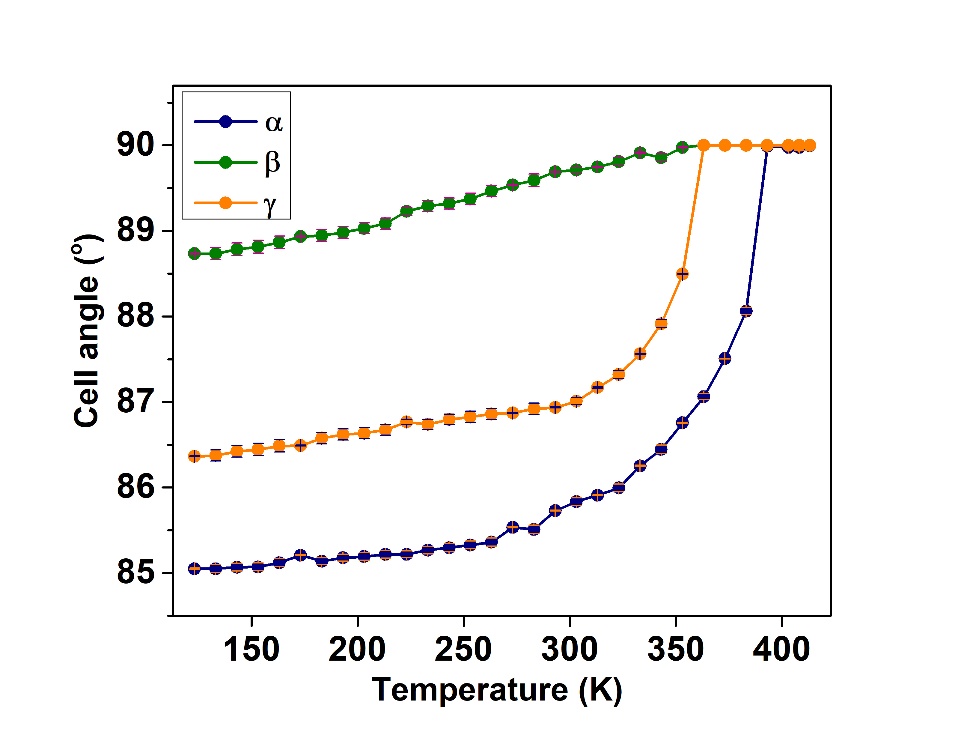


Supplementary Fig. 4 Temperature-dependent cell angles of 1. The crystallographic *β* and *γ* angles rise to 90° at 363 K, implying the space group of crystal 1 changes from triclinic crystal system in the LTP to a monoclinic crystal system in the ITP. Upon further heating, the crystallographic *α* angle becomes 90° at 393 K, implying the orthorhombic crystal system of 1 at the HTP. The different transition behaviors in the two cell angles, *α* and *γ*, verify the triclinic-monoclinic-orthorhombic change of the crystal system.


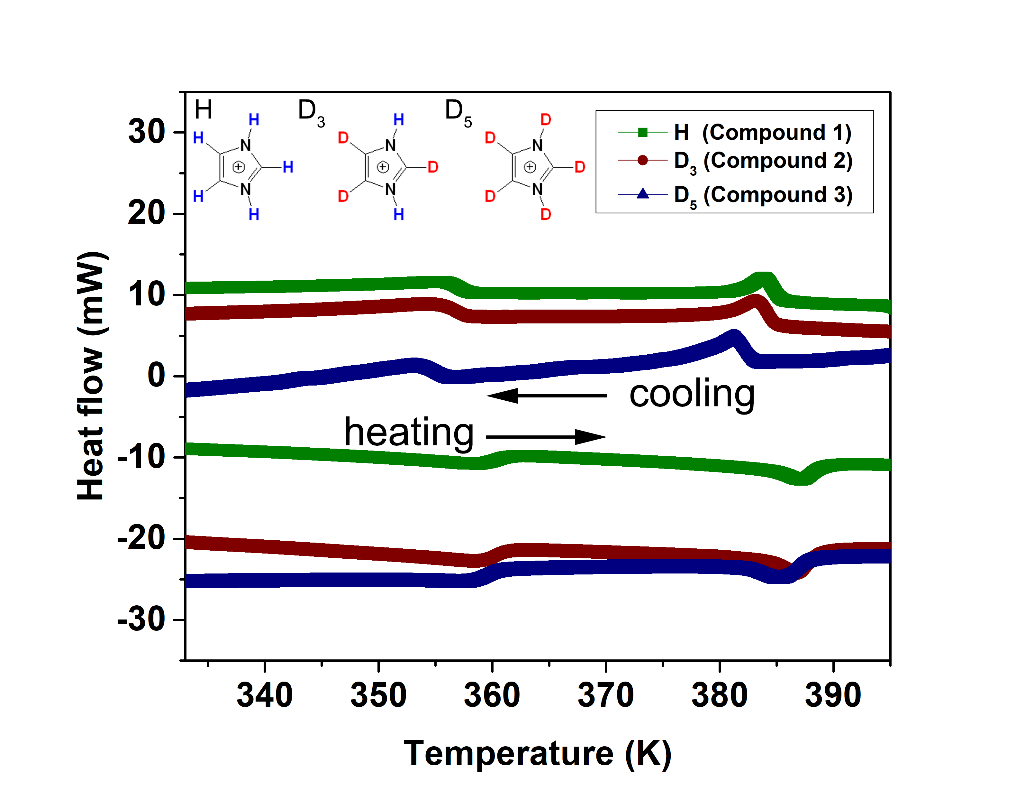


Supplementary Fig. 5 Differential scanning calorimetry of 1, 2, and 3. The two peaks observed in the heating and cooling process indicate that a reversible 2-step phase transition occurs. The deuteration of the sample induces a slight decrease in the phase transition temperatures: *ca.* 0.4 K for compound 2 and *ca.* 1.5 K for compound 3. These values are relatively smaller than those for other hydrogen-bonded phase transition compounds, suggesting that the deuteration has a little effect on the phase transition temperatures of 1^1-4^. The broad anomalous peaks and the narrow hysteresis loop (*ca.* 3 K) suggest that the phase transitions occur in a second-order fashion, which is in agreement with the gradual structural transformation observed in the variable-temperature SCXRD analyses.


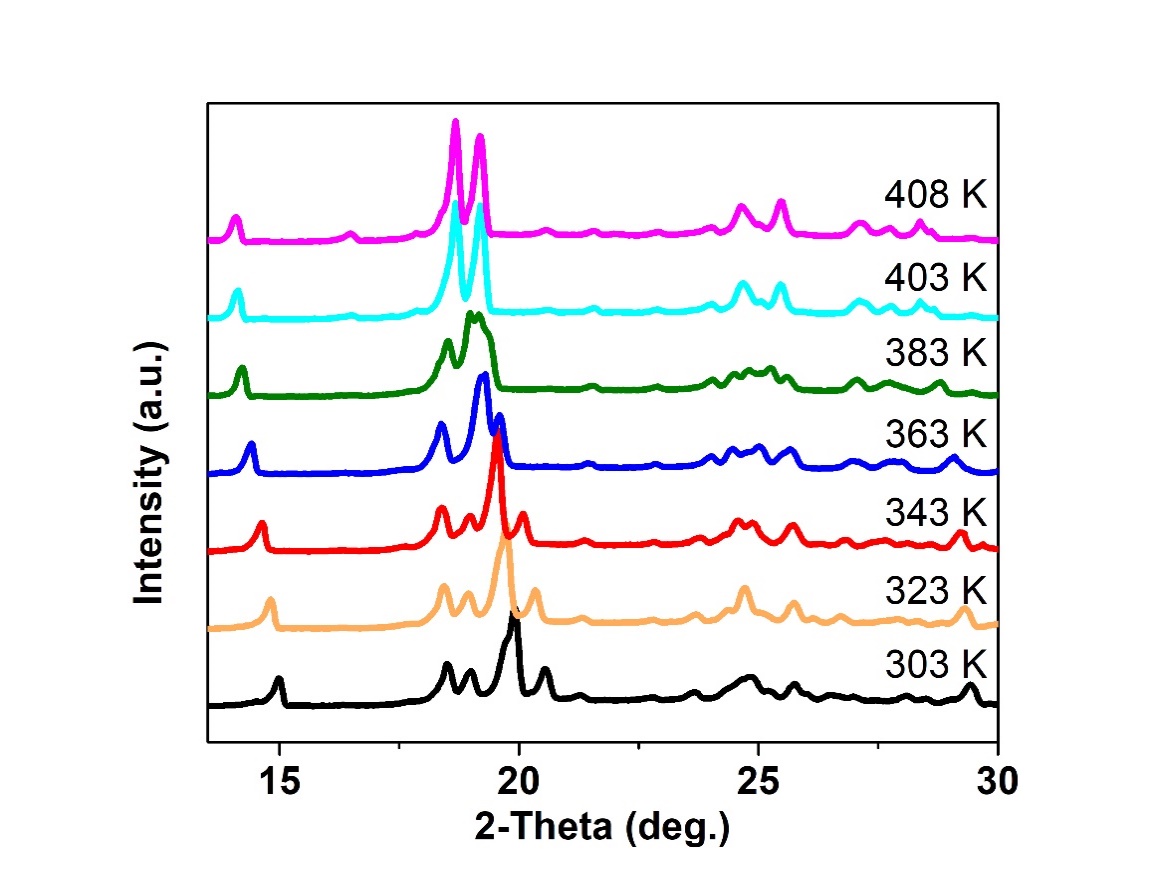


Supplementary Fig. 6 Temperature-dependence of PXRD patterns of 1. The continuous shift of the peak positions suggests the gradual expansion and contraction of the crystal lattice, which is in agreement with the results of SCXRD analyses.


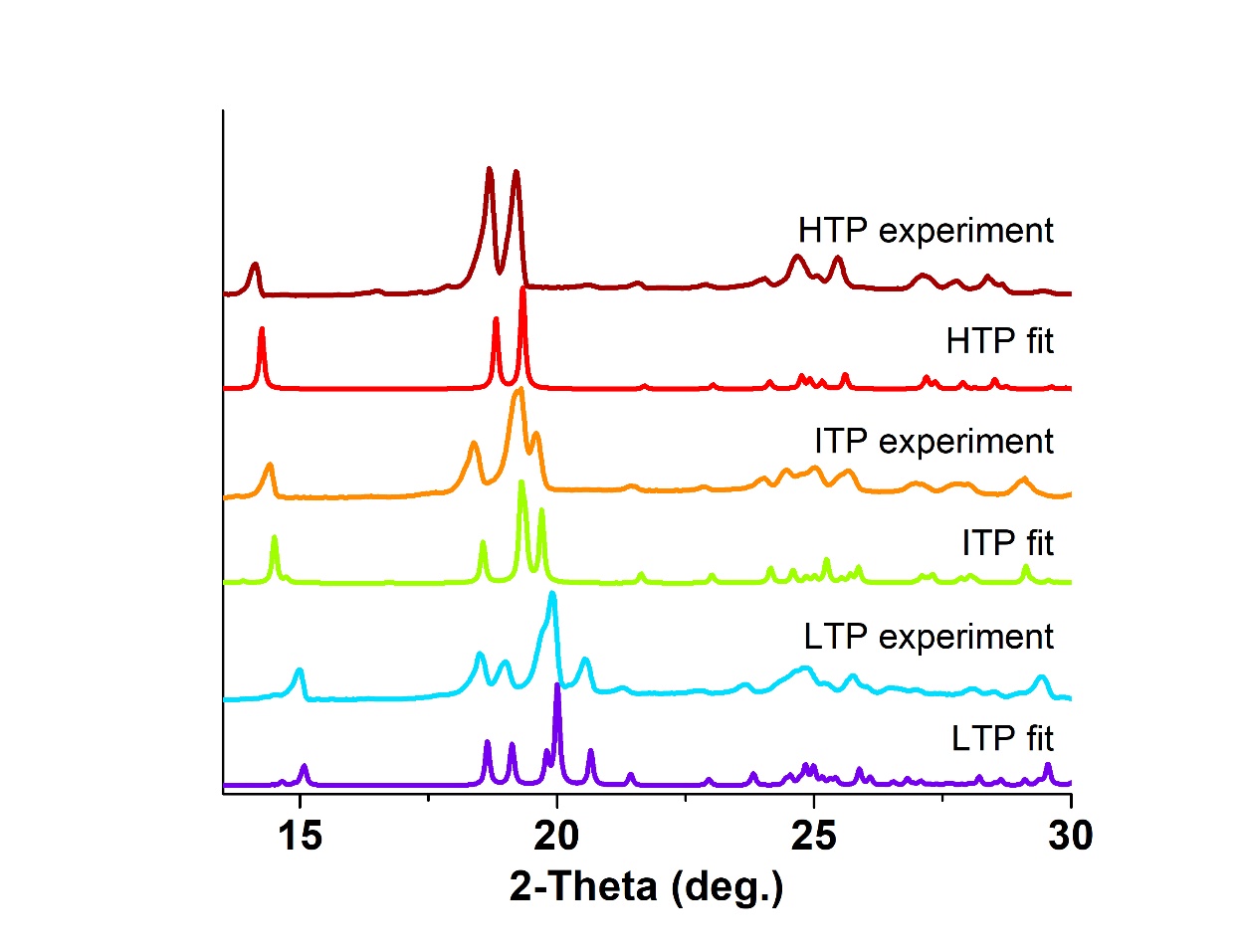


Supplementary Fig. 7 Comparison of the experimental diffraction patterns and the simulated patterns at each phase. The experimental diffraction patterns are in good agreement with the simulated results from SCXRD analyses of each phase, thus confirming the structure and phase purity.


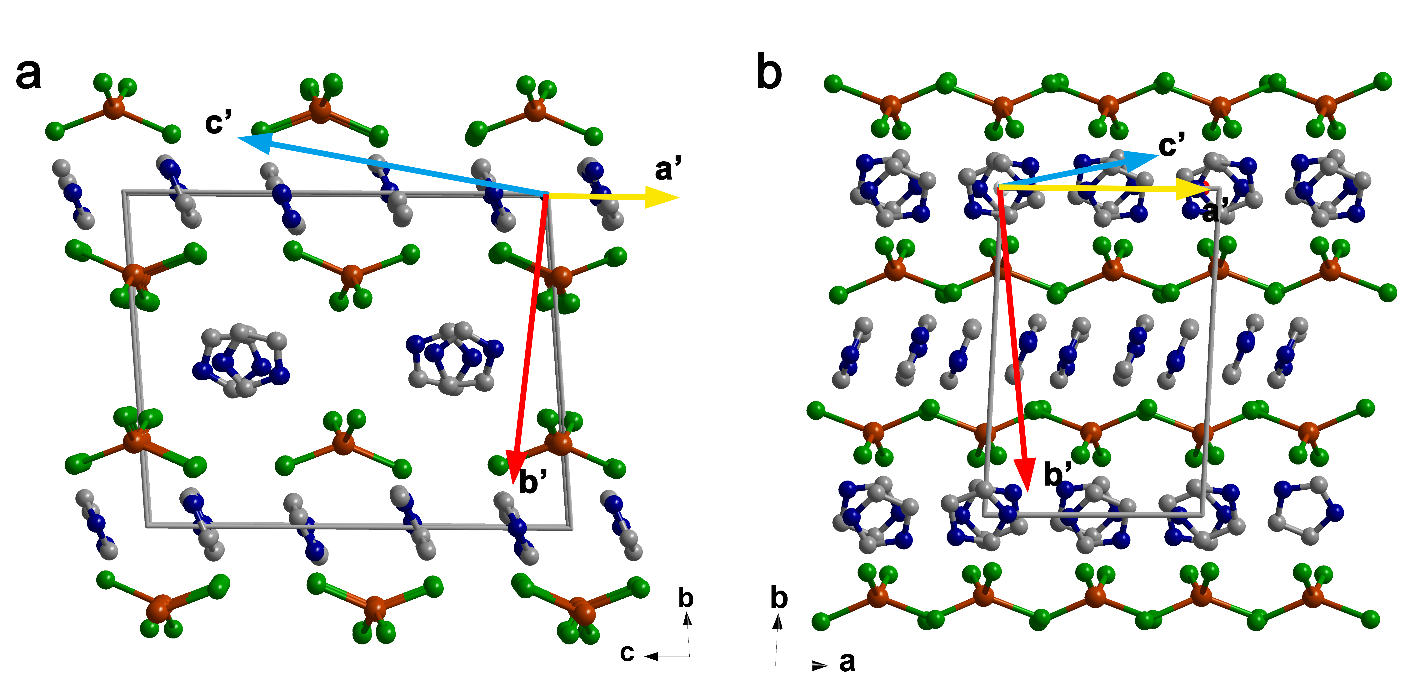


Supplementary Fig. 8 The principal axes of thermal expansion obtained from *PASCal*. The crystal structure is viewed along the crystallographic *a*-axis (a) and *c*-axis (b).

The directions of the principal axes deviate significantly from the crystallographic axes. According to the structural analyses, the NTE along the *a’*-axis is dominated by the reorientation of the Himd^+^ cations in layer B; whereas the NTE along the *c’*-axis is controlled by the reorientation of the Himd^+^ cations in layer A. The larger NTE along the *c’*-axis compared with that along the *a’*-axis, together with the larger increase of *d*_A_ than *d*_B_, suggests the connection mode between the CuCl_4_^2−^ dianions and Himd^+^ cations in layer A is more effective in transmitting the reorientation of molecules to affect global structural changes in materials.


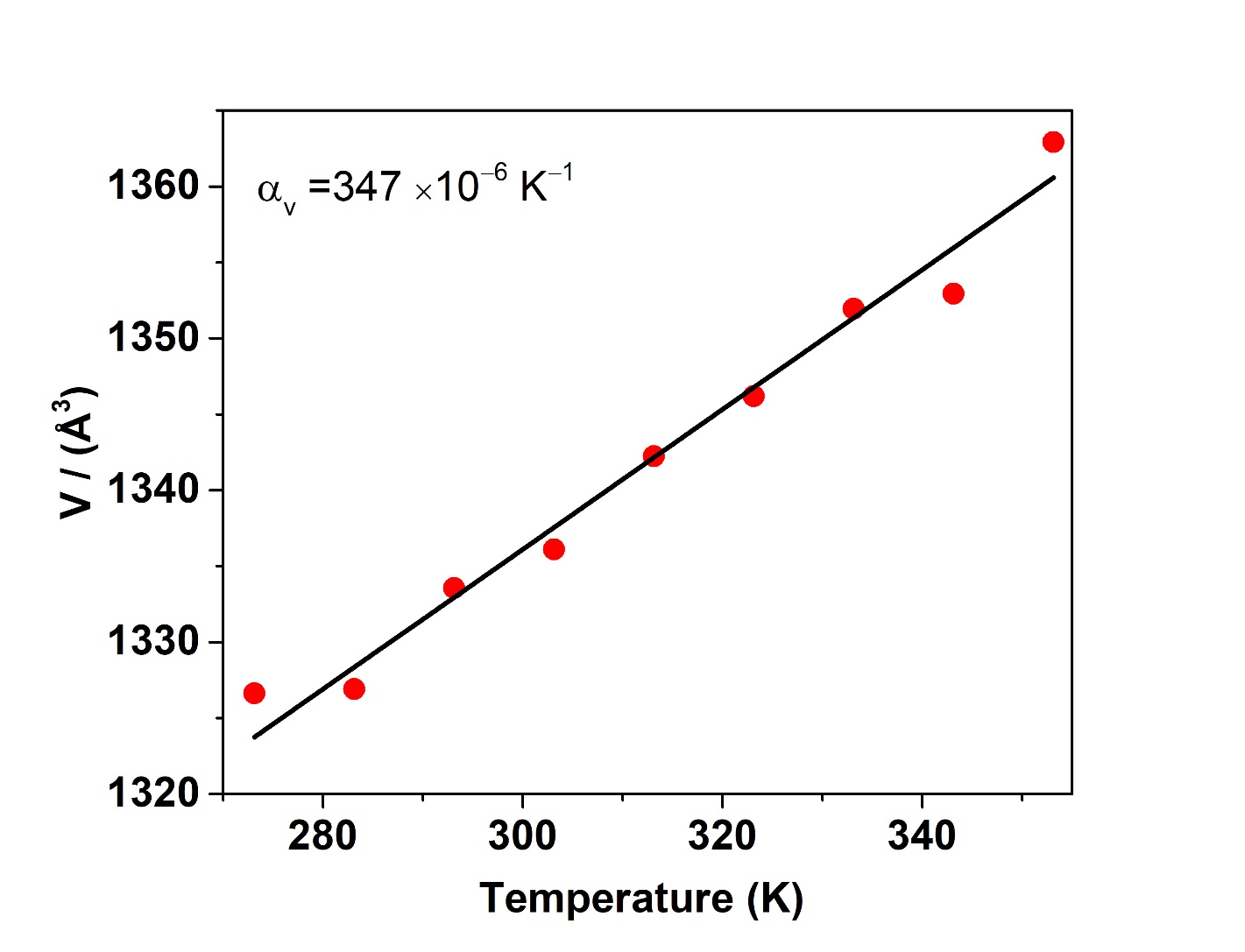


Supplementary Fig. 9 The large volumetric thermal expansion of the crystal in the temperature range from 273 K to 353 K. α_v_ is the volumetric thermal expansion coefficient.


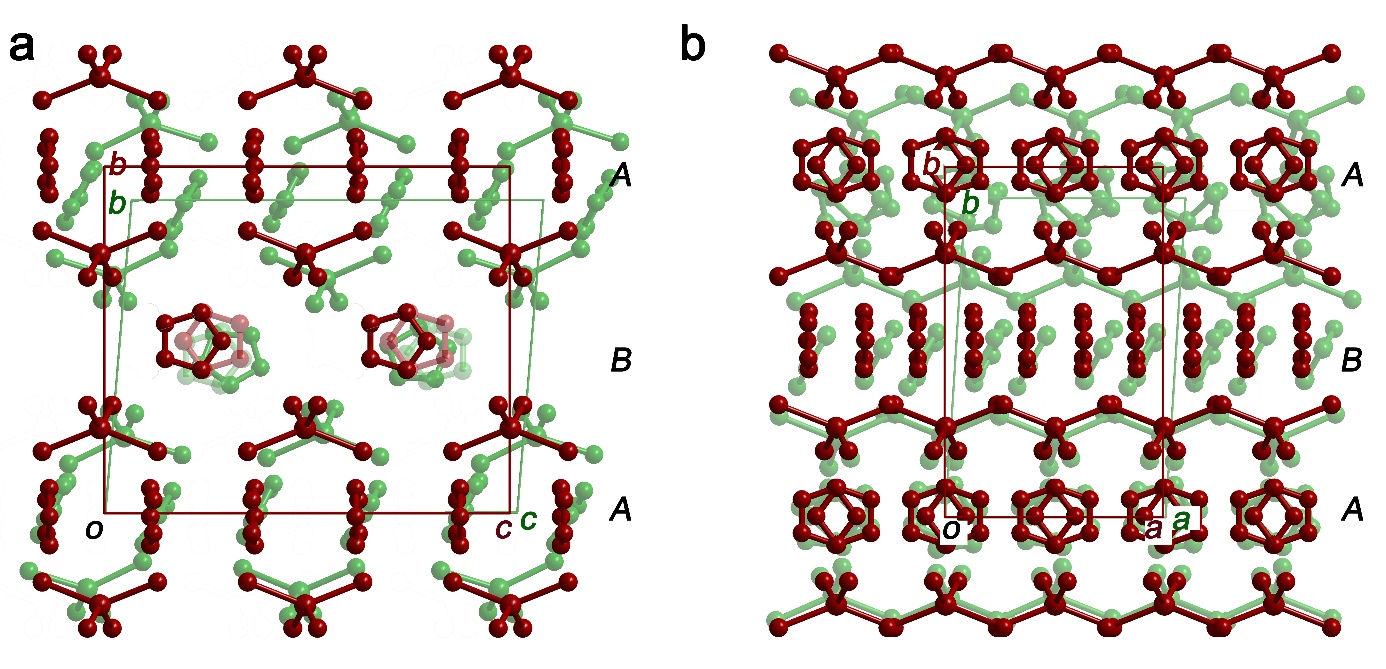


Supplementary Fig. 10 Superimposed drawing of the crystal structures in the HTP (393 K, red) and LTP (123 K, green). The crystal structure is viewed along the crystallographic *a*-axis (a) and *c*-axis (b). The panoramic view of crystal structure reveals that the reorientation of molecular cations actuates a large expansion in the interlayer distance of CuCl_4_^2−^ dianions and induces a significant sliding movement of the layers in the crystallographic (010) plane. Notably, the orientational change in dianions is much smaller than that in the molecular cations with respect to the crystallographic (010) plane. The remarkable displacive motion of dianions suggests that the CuCl_4_^2−^ ions play important roles in transmitting the motion of molecular cations into a giant anisotropic thermal expansion of the bulk crystal.


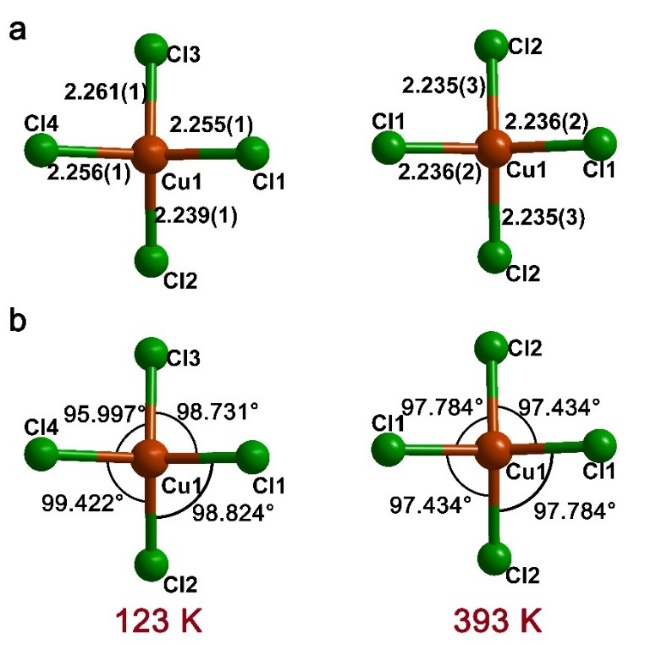


**Supplementary Fig. 11** The molecular structure of CuCl_4_^2−^ dianions. The large structural transformation of crystal induces a small change in the Cu−Cl bond lengths (**a**) and Cl−Cu−Cl angles (**b**).


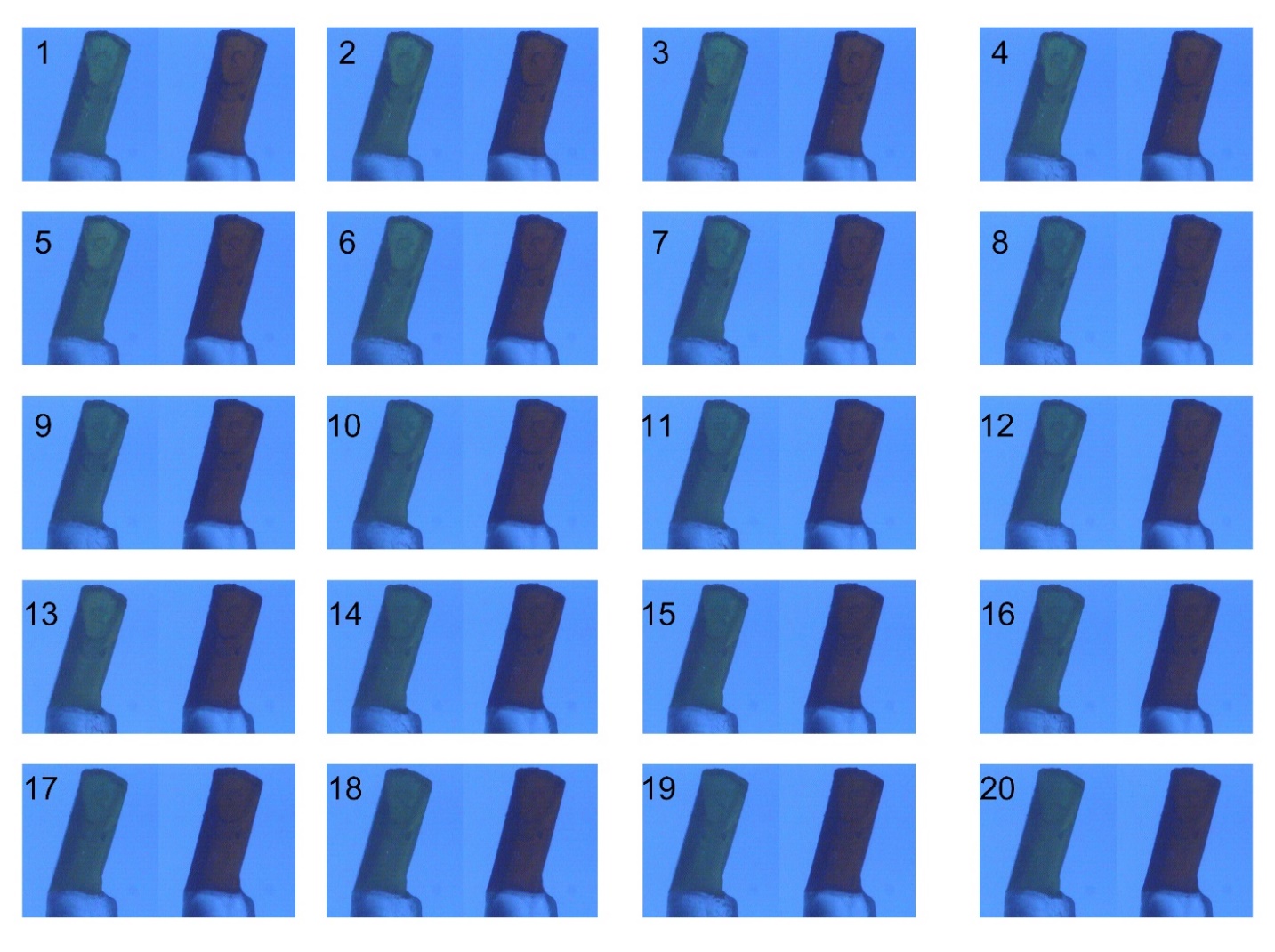


Supplementary Fig. 12 Reversible shrinkage and expansion in the crystal thickness upon cooling and heating at a rate of 20 K min^−1^ in the temperature range of 123–403 K. The reversible crystal shape change was recorded for 20 cycles without perceptible deterioration in the crystal quality. Notably, the structural transition also leads to a yellow to red color change of the single crystal upon heating. However, no substantial geometric transformation was identified in the complex anions of CuCl_4_^2−^. Hence, the color change of the single crystal could be attributed to the reorientation of molecules, as observed in the other reported compounds^5,6^.

**
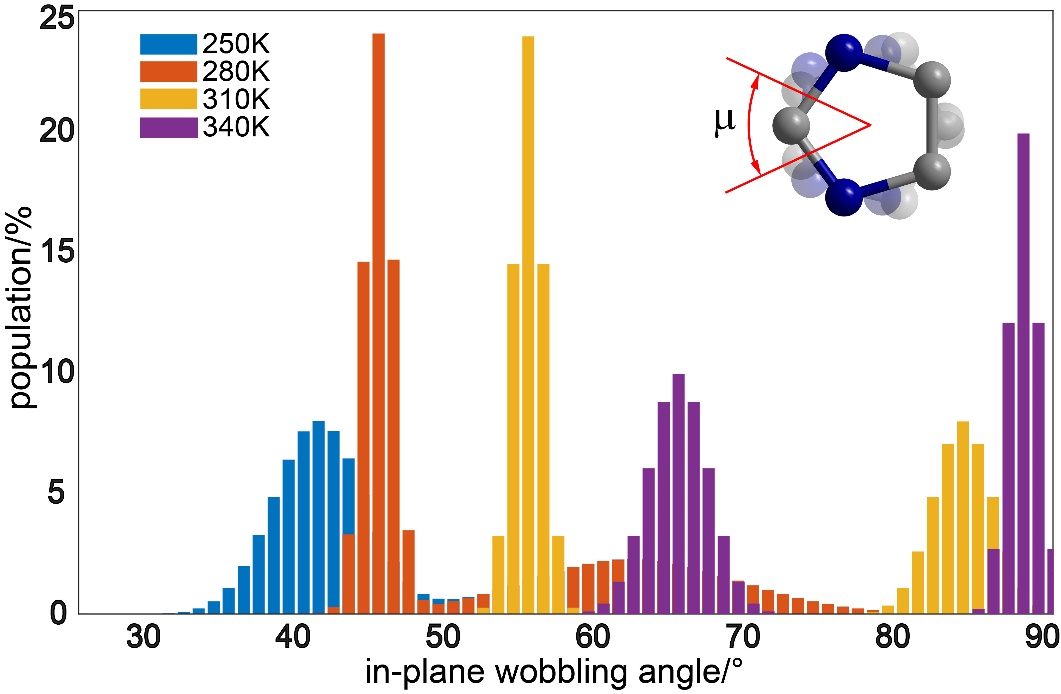
**

Supplementary Fig. 13 The distribution of in-plane wobbling angles of Himd^+^ cations at the LTP. The wobbling amplitude (angle *μ*) was significantly intensified as the temperature increases.

Supplementary Fig. 14 The ^2^H NMR spectra obtained from 310 K to 370 K. The component of full rotation appears between 350 K to 360 K, as seen from the Pake pattern with quadrupolar splitting of 60 kHz.


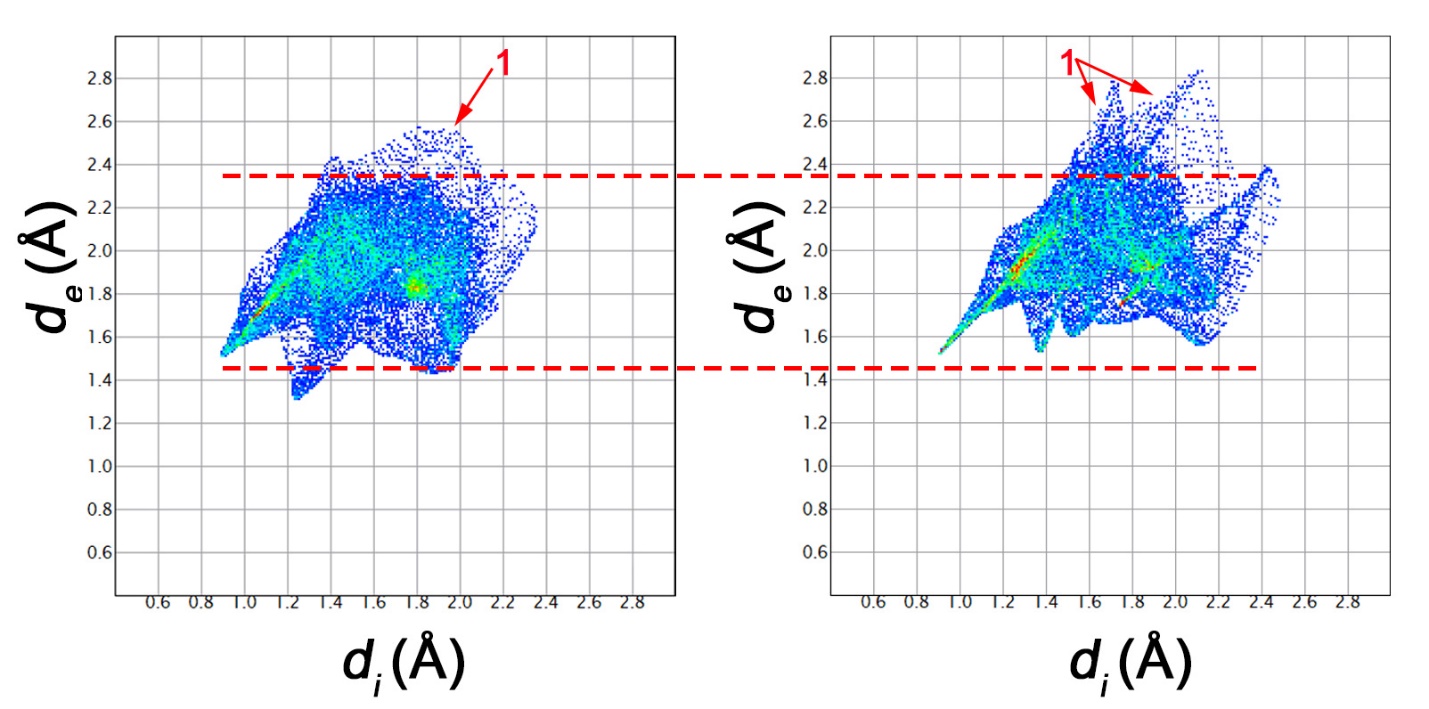


Supplementary Fig. 15 Fingerprint plots generated from Hirshfeld surfaces of Himd^+^ cation in layer A. The fingerprint plots at the HTP (right) shift significantly to the region with larger *d*_e_ in contrast to those in the LTP (left), suggesting a weaker molecular interactions in the HTP^7^. Moreover, the diffuse blue region (labelled 1), which results from a small part of the surface with large distances to the nearest atoms, was observed in both the fingerprint plots of the LTP and HTP, indicating a loose-packed nature of superstructure. Notably, the diffusion region in the HTP is more prominent than that in the LTP, suggesting a larger free volume around the Himd^+^ in the HTP.


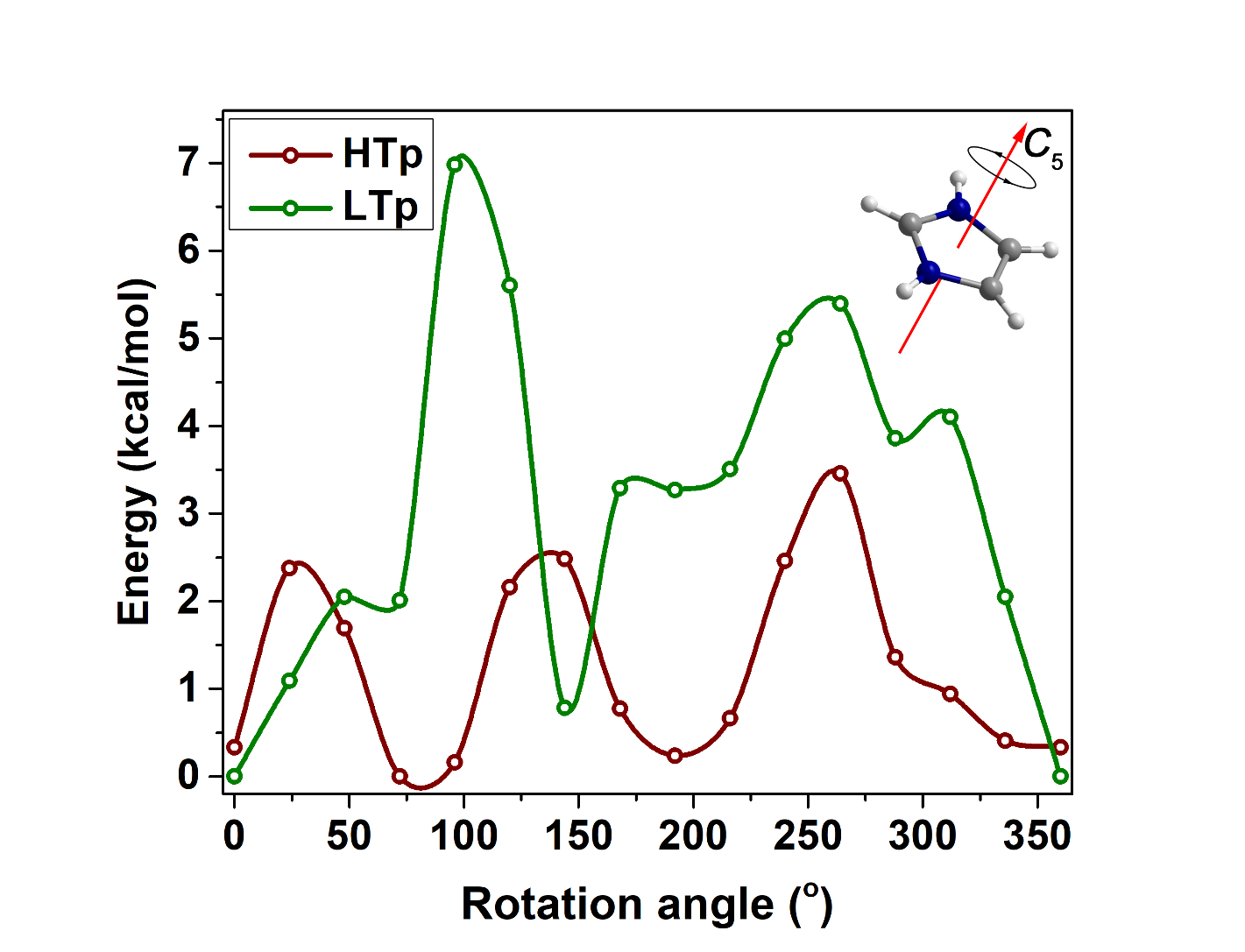


Supplementary Fig. 16 Potential energy curves for 360º rotation of the Himd^+^ cation along its *C*_5_-axis. The variation of temperature induces a significant change in the potential energy for the rotation of Himd^+^ cation along its molecular *C*_5_-axis.
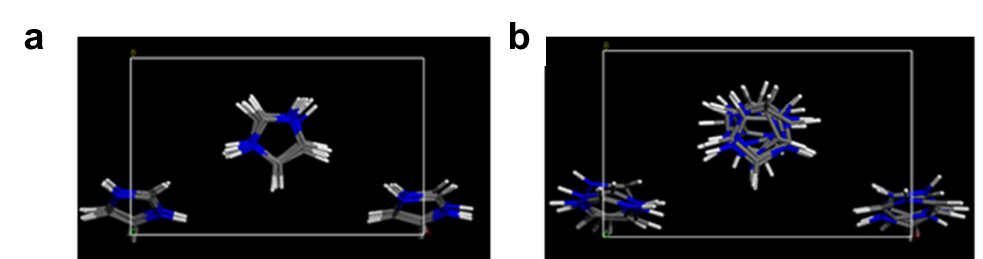


Supplementary Fig. 17 The overlap of molecular cations found in the molecular dynamic simulations performed at 123 K (a) and 413 K (b). In contrast to the in-plane wobbling found at 123 K (LTP), the molecular cations demonstrate a heavily rotation around the molecular pseudo *C*_5_-axis at 413 K (HTP).


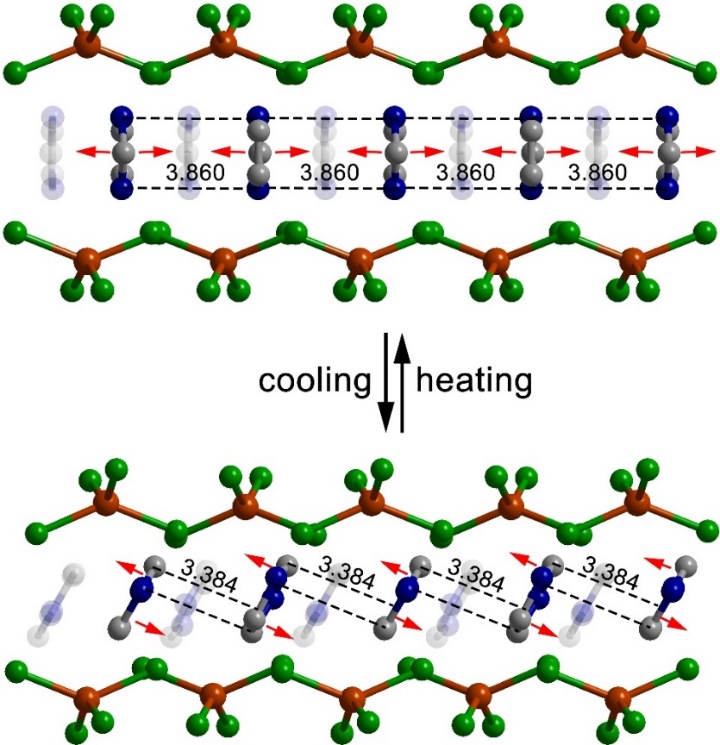


**Supplementary Fig. 18** The electrostatic repulsions between the molecular cations in layer B. The red arrows denote the dominating electrostatic repulsions on the molecular cations. The molecular cations in different crystallographic face are drawn in transparency. The unbalanced electrostatic repulsions on molecular planes of cations in layer B are enhanced because the Himd^+^ cations in layer B stack in a one-dimensional offset column in contrast to the dimer structure of molecular cations in layer A. The enhancement of unbalanced electrostatic repulsions should play substantial roles in the reorientation of molecular cations in layer B in the ITP. Furthermore, the strong electrostatic repulsions between cations are confirmed by the significant increase in interplanar distances of molecular cations, which elongate from 3.393/3.498 Å (layer A) and 3.384 Å (layer B) in the LTP (bottom) to 3.704 Å and 3.860 Å in the HTP (top), respectively.

The DFT calculations revealed that the repulsive energy between a pair of Himd^+^ cations in layer A increases from 69.6 kcal/mol at the LTP to 71.6 kcal/mol at the HTP, suggesting the co-planar packing of Himd^+^ cations at the HTP is unstable, and should change to an enthalpically favorable offset-packing mode at the LTP.
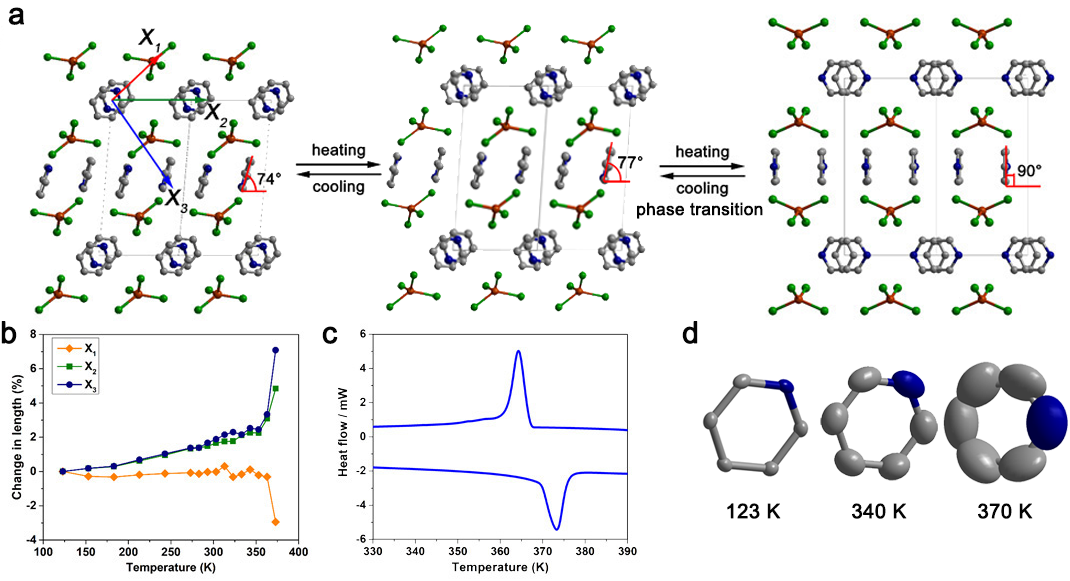


Supplementary Fig. 19 The large anisotropic thermal expansion of the single crystal Bis(pyridinium) tetrachlorocuprate (4) actuated by a thermodynamically assisted reorientation of pyridinium cations. a, The crystal structures of 3 in the low-temperature phase (left: 123 K; middle: 340 K) and the high-temperature phase (right: 370 K). The dihedral angles between the pyridinium cations and crystallographic (001) face shift from 74° at 123 K to 90° at 370 K accompanied with the statically ordered-rotationally disordered change in molecular cations. The reorientation of the cations induces a large thermal expansion of the single crystal with the directions of principal axes denoted in the left panel. b, The anisotropic thermal expansion obtained from *PASCal* program along the principal axes. As the dihedral angle between the pyridine plane and the crystallographic (001) plane shifts from 74° at 123 K to 90° at 373 K, a *ca.* 7% expansion of the crystal along its principal axis was detected. c, The DSC curve of crystal. The sharp anomaly peaks and wide thermal hysteresis loop (*ca.* 11 K) indicate the first order phase transition of 4. d, The thermal vibrations of pyridinium at different temperatures. The large thermal ellipsoids of the cations suggest the pyridinium cations are rotationally disordered at a high-temperature phase.


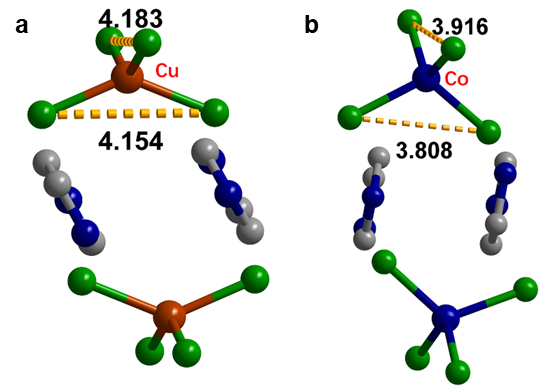


Supplementary Fig. 20 Comparison of the tetrahedral geometry of complex anion in the sample 1 (a) and 5 (b). The Jahn-Teller effect of Cu(II) metal center induces a significant flattening in the tetrahedral geometry of the Cu(II) complex anion. Correspondingly, the Cl−M−Cl structure that encloses the molecular cations demonstrates a large variation when the Cu(II) was substituted with Co(II), *i.e,* the Cl···Cl distance decreases from 4.2 Å for Cu(II) complex to 3.9 Å for Co(II) complex. We suppose such structural transformation accounts for the difference thermal response of materials.


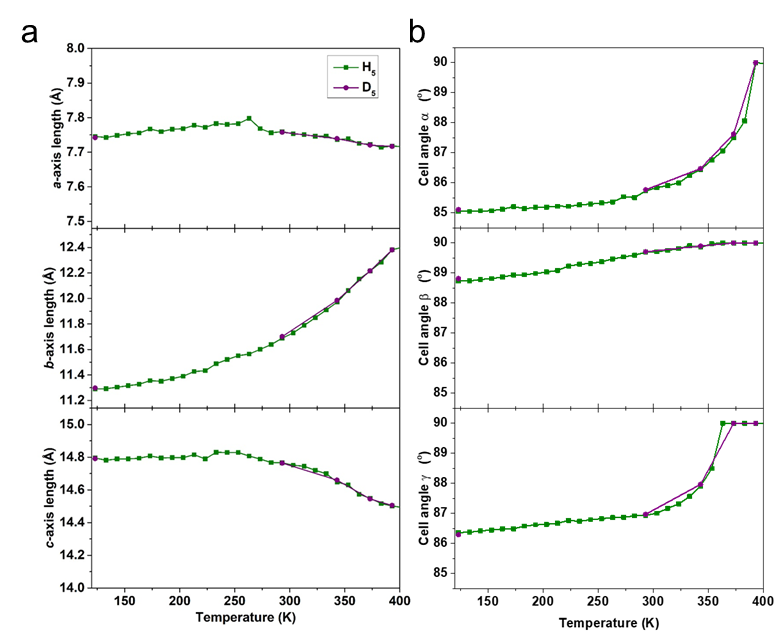


**Supplementary Fig. 21** Comparison of the temperature-dependent cell lengths (**a**) and angles (**b**) of compound **1** and **3**. The deuterated analog has very similar cell parameters with that of compound **1**.


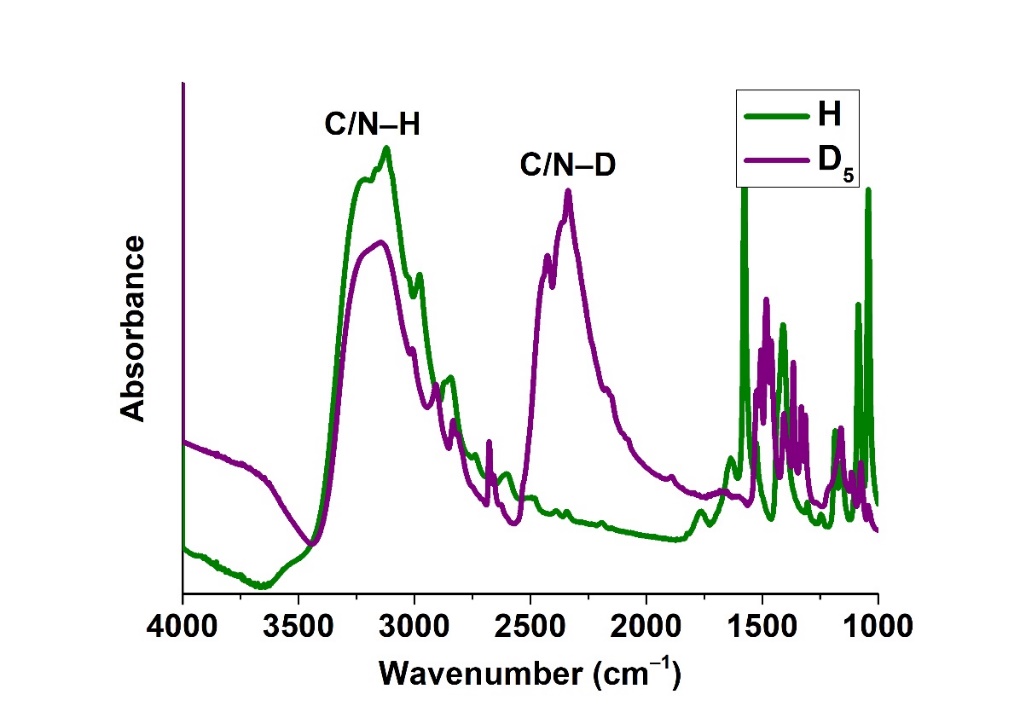


**Supplementary Fig. 22** IR spectra of bis(imidazolium) tetrachlorocuprate **(1)** and bis(*D*_5_-imidazolium) tetrachlorocuprate **(3)**.

**Supplementary Table 1** Crystallographic parameters for complex **1**.

|  | **LTP** | | | | | | |
| --- | --- | --- | --- | --- | --- | --- | --- |
| Temperature/K | 123 | 173 | 223 | 273 | 293 | 313 | 333 |
| Empirical formula | C_12_H_20_Cl_8_Cu_2_N_8_ | | | | | | |
| Space group | *P-*1 | *P-*1 | *P-*1 | *P-*1 | *P-*1 | *P-*1 | *P-*1 |
| *a*/Å | 7.758(2) | 7.765(2) | 7.765(2) | 7.7693(17) | 7.765(2) | 7.753(3) | 7.776(5) |
| *b*/Å | 11.291(3) | 11.350(3) | 11.456(3) | 11.599(3) | 11.688(3) | 11.779(3) | 11.936(7) |
| *c*/Å | 14.813(4) | 14.802(4) | 14.810(4) | 14.800(3) | 14.778(3) | 14.746(5) | 14.713(10) |
| *α*/° | 85.090(9) | 85.116(7) | 85.309(7) | 85.505(6) | 85.655(8) | 85.906(7) | 86.31(2) |
| *β*/° | 88.829(10) | 89.031(9) | 89.307(6) | 89.476(7) | 89.657(9) | 89.790(7) | 89.90(2) |
| *γ*/° | 86.337(9) | 86.438(7) | 86.723(7) | 86.863(6) | 86.939(6) | 87.157(8) | 87.53(2) |
| Volume/Å^3^ | 1290.1(6) | 1297.3(7) | 1310.8(6) | 1327.6(5) | 1335.4(6) | 1341.6(7) | 1361.6(15) |
| Dcalc./mg·m^−3^ | 1.769 | 1.759 | 1.741 | 1.719 | 1.709 | 1.701 | 1.676 |
| *μ*/mm^−1^ | 2.495 | 2.481 | 2.455 | 2.424 | 2.410 | 2.399 | 2.364 |
| *F*_000_ | 684 | 684 | 684 | 684 | 684 | 684 | 684 |
| Reflections collected | 10641 | 18584 | 10869 | 18966 | 17658 | 16500 | 18861 |
| Independent reflections | 5783 | 5919 | 5753 | 6030 | 6044 | 6095 | 6117 |
| *R*(int) | 0.0345 | 0.0410 | 0.0326 | 0.0418 | 0.0590 | 0.0486 | 0.0510 |
| Completeness | 98.7% | 99.5% | 97.7% | 99.5% | 99.2% | 99.5 % | 99.4% |
| Data/restraints/parameters | 5783/0/271 | 5919/0/271 | 5753/0/271 | 6030/0/271 | 6044/0/271 | 6095/0/271 | 6117/0/271 |
| Goodness-of-fit on *F*^2^ | 0.831 | 1.032 | 0.963 | 1.014 | 0.775 | 0.880 | 0.812 |
| *R_1_*^a^ [I > 2σ(I)] | 0.0407 | 0.0423 | 0.0400 | 0.0403 | 0.0389 | 0.0447 | 0.0428 |
| *ωR_2_*^b^(all data) | 0.1420 | 0.1534 | 0.1051 | 0.1020 | 0.0884 | 0.1274 | 0.1267 |

|  | **LTP** | **ITP** | **HTP** | |
| --- | --- | --- | --- | --- |
| Temperature/K | 353 | 373 | 393 | 413 |
| Empirical formula | C_12_H_20_Cl_8_Cu_2_N_8_ | | | |
| Space group | *P-*1 | *P*2_1_/*c* | *Pcma* | *Pcma* |
| *a*/Å | 7.771(4) | 12.215(2) | 7.718(2) | 7.7157(16) |
| *b*/Å | 12.083(7) | 7.7229(14) | 12.382(4) | 12.415(2) |
| *c*/Å | 14.689(8) | 14.548(3) | 14.502(5) | 14.483(3) |
| *α*/° | 86.636(19) | 90 | 90 | 90 |
| *β*/° | 89.86(2) | 92.495(5) | 90 | 90 |
| *γ*/° | 88.451(15) | 90 | 90 | 90 |
| Volume/Å^3^ | 1376.5(13) | 1371.1(4) | 1385.9(7) | 1387.3(5) |
| Dcalc./mg·m^−3^ | 1.658 | 1.664 | 1.646 | 1.645 |
| *μ*/mm^−1^ | 2.338 | 2.347 | 2.322 | 2.320 |
| *F*_000_ | 684 | 684 | 684 | 684 |
| Reflections collected | 15646 | 2684 | 1351 | 16220 |
| Independent reflections | 6157 | 2684 | 1351 | 1324 |
| *R*(int) | 0.0556 | 0.0289 | 0.0438 | 0.0404 |
| Completeness | 99.2% | 99.7% | 99.7% | 99.9% |
| Data/restraints/parameters | 6157/0/271 | 2684/0/136 | 1351/0/72 | 1324/0/72 |
| Goodness-of-fit on *F*^2^ | 0.824 | 0.784 | 1.180 | 1.262 |
| *R_1_*^a^ [I > 2σ(I)] | 0.0506 | 0.0430 | 0.0711 | 0.0866 |
| *ωR_2_*^b^(all data) | 0.1602 | 0.0896 | 0.1973 | 0.2028 |
| ^a^ *R_1_* = Σ\|\|*F*_o_\|-\|*F*_c_\|\|/Σ\|*F*_o_\|. ^b^ *ωR_2_* = {Σ[ω(*F*_o_^2^-*F*_c_^2^)^2^]/Σ[ω(*F*_o_^2^)^2^]}^½^ | | | | |

Notably, the crystallographic a and b axes were exchanged at the ITP of 373 K.

During the two-step phase transition, the symmetric elements of **1** increase from two (*E*, *i*) in the LTP to four (*E*, *C*_2_, *i*, and *σ_h_*) in the ITP, and then change to eight (*E*, 3*C*_2_, *i*, *σ_v_*, *σ_d_*_,_ and *σ_h_*) in the HTP, which are consistent with two symmetry breaking ferroelastic-to-paraelastic phase transitions with Aizu notations of 2/*m*F−1 and *mmm*F2/*m*, respectively^8^.

**Supplementary Table 2** Comparison of thermal expansion coefficients for selected materials.

| Compound | *T* (K) | *α*_PTE_  (× 10^−6^ K^-1^) | *α*_NTE_  (× 10^−6^ K^-1^) | Reference |
| --- | --- | --- | --- | --- |
| Ag_3_Co(CN)_6_ | 10~500 | 132 | −130 | 9 |
| ZrW_2_O_8_ | 0.3~1050 | NA | −9.1 | 10 |
| [Ag(en)]NO_3_-I | 120~360 | 149 | −90 | 11 |
| Cd(im) | 100~300 | 93 | −23 | 12 |
| Ag(mim) | 20~300 | 130 | −25 | 13 |
| [Zn(OH)(niba)]∙MeOH | 100~370 | 166 | NA | 14 |
| HMOF-1 | 100~380 | 177 | NA | 15 |
| FMOF-1 | 90~295 | 230 | −170 | 16 |
| MCF-18∙MeOH | 119~295 | 81~437 | NA | 17 |
| MCF-34 | 127~673 | 224 | −107 | 18 |
| MCF-82 | 112~300 | 482 | −218 | 19 |
| [(H_4_BPTC) (BPE)_2_] | 123~408 | 125~394 | NA | 20 |
| FJ1-H11-Me | 100~293 | 653 | −38 | 21 |
| 2(4PazP)∙(4,6-diCl res) | 260~290 | 316 | −116 | 22 |
| aspirin | 93~323 | 93 | NA | 23 |
| (S,S)-Octa-3,5-diyn-2,7-dio | 225~330 | 156~515 | −85 | 6 |
| PHA-*α* | 223~348 | 260 | −80 | 24 |
| CD_3_OD·D_2_O | 40~160 | 462 | −61 | 25 |
| 1 | 273~353 | 568 | −184 | This work |

**Supplementary Table 3** Crystallographic parameters for crystal **2**.

|  | LTP | LTP | ITP | HTP |
| --- | --- | --- | --- | --- |
| Temperature/K | 123 | 293 | 360 | 395 |
| Empirical formula | C_12_D_12_Cl_8_Cu_2_H_8_N_8_ | | | |
| Formula weigh | 699.11 | | | |
| Crystal size/mm | 0.150 × 0.060× 0.050 | | | |
| Space group | *P-*1 | *P-*1 | *P*2_1_/*c* | *Pbam* |
| *a*/Å | 7.7651(3) | 7.7717(8) | 12.1719(15) | 7.7334(12) |
| *b*/Å | 11.2850(3) | 11.7264(11) | 7.7394(9) | 14.504(3) |
| *c*/Å | 14.8232(5) | 14.7791(14) | 14.6148(12) | 12.396(3) |
| *α*/° | 85.059(2) | 85.692(8) | 90 | 90 |
| *β*/° | 88.682(3) | 89.727(8) | 92.860(10) | 90 |
| *γ*/° | 86.299(3) | 87.188(8) | 90 | 90 |
| Volume/Å^3^ | 1291.22(7) | 1334.7(4) | 1375.1(3) | 1390.4(5) |
| Z | 2 | 2 | 2 | 2 |
| Dcalc./mg·m^−3^ | 1.798 | 1.731 | 1.689 | 1.670 |
| *μ*/mm^−1^ | 2.492 | 2.399 | 2.340 | 2.315 |
| *F*_000_ | 684 | 684 | 684 | 684 |
| Reflections collected | 20833 | 18957 | 6970 | 8150 |
| Independent reflections | 4662 | 4841 | 3231 | 1785 |
| *R*(int) | 0.0464 | 0.0960 | 0.0433 | 0.0328 |
| Completeness | 99.5% | 99.7% | 96.4% | 98.1% |
| Data/restraints/parameters | 4662/0/271 | 4841/0/271 | 3231/0/136 | 1785/0/72 |
| Goodness-of-fit on *F*^2^ | 0.942 | 1.205 | 0.955 | 0.911 |
| *R_1_*^a^ [I > 2σ(I)] | 0.0341 | 0.0823 | 0.0622 | 0.0477 |
| *ωR_2_*^b^(all data) | 0.1196 | 0.2197 | 0.2089 | 0.2062 |
| ^a^ *R_1_* = Σ\|\|*F*_o_\|-\|*F*_c_\|\|/Σ\|*F*_o_\|. ^b^ *ωR_2_* = {Σ[ω(*F*_o_^2^-*F*_c_^2^)^2^]/Σ[ω(*F*_o_^2^)^2^]}^½^ | | | | |

**Supplementary Table 4** Crystallographic parameters for crystal **3**.

|  | LTP | LTP | ITP | HTP |
| --- | --- | --- | --- | --- |
| Temperature/K | 123 | 293 | 373 | 393 |
| Empirical formula | C_12_D_20_Cl_8_Cu_2_N_8_ | | | |
| Formula weigh | 707.16 | | | |
| Crystal size/mm | 0.150 × 0.130× 0.080 | | | |
| Space group | *P-*1 | *P-*1 | *P*2_1_/*c* | *Pbam* |
| *a*/Å | 7.7421(12) | 7.7577(15) | 12.216(2) | 7.717(2) |
| *b*/Å | 11.2979(17) | 11.701(2) | 7.7205(14) | 14.505(4) |
| *c*/Å | 14.791(2) | 14.764(3) | 14.545(3) | 12.380(3) |
| *α*/° | 85.111(5) | 85.769(7) | 90 | 90 |
| *β*/° | 88.815(5) | 89.707(6) | 92.377(5) | 90 |
| *γ*/° | 86.290(5) | 86.973(6) | 90 | 90 |
| Volume/Å^3^ | 1286.2(3) | 1334.7(4) | 1370.6(4) | 1385.8(6) |
| Z | 2 | 2 | 2 | 2 |
| Dcalc./mg·m^−3^ | 1.826 | 1.760 | 1.714 | 1.695 |
| *μ*/mm^−1^ | 2.502 | 2.411 | 2.348 | 2.322 |
| *F*_000_ | 684 | 684 | 684 | 684 |
| Reflections collected | 11455 | 21454 | 11890 | 8596 |
| Independent reflections | 5168 | 7391 | 2833 | 1291 |
| *R*(int) | 0.0484 | 0.0797 | 0.0577 | 0.0530 |
| Completeness | 98.5% | 99.7% | 99.6% | 99.9% |
| Data/restraints/parameters | 5168/0/271 | 7391/0/271 | 2833/0/136 | 1291/0/73 |
| Goodness-of-fit on *F*^2^ | 1.131 | 1.210 | 1.169 | 0.643 |
| *R_1_*^a^ [I > 2σ(I)] | 0.0589 | 0.0838 | 0.0959 | 0.0724 |
| *ωR_2_*^b^(all data) | 0.1659 | 0.2079 | 0.2526 | 0.2739 |
| ^a^ *R_1_* = Σ\|\|*F*_o_\|-\|*F*_c_\|\|/Σ\|*F*_o_\|. ^b^ *ωR_2_* = {Σ[ω(*F*_o_^2^-*F*_c_^2^)^2^]/Σ[ω(*F*_o_^2^)^2^]}^½^ | | | | |

**Supplementary Table 5** Crystallographic parameters for crystal **4**.

| Temperature/K | 123 | 340 | 370 |
| --- | --- | --- | --- |
| Empirical formula | C_5_H_6_Cl_2_Cu_0.5_N | | |
| Formula weigh | 182.78 | | |
| Crystal size/mm | 0.22 × 0.1 × 0.08 | | |
| Space group | *C*2/*c* | *C*2/*c* | *Cccm* |
| *a*/Å | 7.9167(5) | 8.0048(5) | 8.108(3) |
| *b*/Å | 13.5682(8) | 13.9122(9) | 14.272(3) |
| *c*/Å | 13.4123(7) | 13.5860(9) | 13.630(3) |
| *α*/° | 90 | 90 | 90 |
| *β*/° | 95.595(5) | 94.259(7) | 90 |
| *γ*/° | 90 | 90 | 90 |
| Volume/Å^3^ | 1433.82(15) | 1508.82(17) | 1577.2(7) |
| Z | 8 | 8 | 8 |
| Dcalc./mg·m^−3^ | 1.693 | 1.609 | 1.54 |
| *μ*/mm^−1^ | 2.247 | 2.135 | 2.043 |
| *F*_000_ | 732 | 732 | 732 |
| Reflections collected | 5907 | 8864 | 3955 |
| Independent reflections | 1797 | 1930 | 722 |
| *R*(int) | 0.0334 | 0.0433 | 0.0843 |
| Completeness | 99.5% | 99.5% | 96.1% |
| Data/restraints/parameters | 1797/0/78 | 1930/0/78 | 722/0/43 |
| Goodness-of-fit on *F*^2^ | 1.103 | 0.921 | 1.112 |
| *R_1_*^a^ [I > 2σ(I)] | 0.0288 | 0.0358 | 0.0947 |
| *ωR_2_*^b^(all data) | 0.0782 | 0.1218 | 0.2900 |
| ^a^ *R_1_* = Σ\|\|*F*_o_\|-\|*F*_c_\|\|/Σ\|*F*_o_\|. ^b^ *ωR_2_* = {Σ[ω(*F*_o_^2^-*F*_c_^2^)^2^]/Σ[ω(*F*_o_^2^)^2^]}^½^ | | | |

**Supplementary Table 6** Crystallographic parameters for crystal **5**.

| Temperature/K | 123 | 390 |
| --- | --- | --- |
| Empirical formula | C_6_H_10_Cl_4_CoN_4_ | |
| Formula weigh | 338.91 | |
| Crystal size/mm | 0.20 × 0.18 × 0.16 | |
| Space group | *P*2_1_/*c* | *C*2/*c* |
| *a*/Å | 7.5432(4) | 8.0002(15) |
| *b*/Å | 13.5866(5) | 13.384(2) |
| *c*/Å | 12.7767(6) | 13.003(3) |
| *α*/° | 90 | 90 |
| *β/*° | 93.296(4) | 91.680(18) |
| *γ*/° | 90 | 90 |
| Volume/Å^3^ | 1307.27(10) | 1391.7(5) |
| Z | 4 | 4 |
| Dcalc./mg·m^−3^ | 1.722 | 1.618 |
| *μ*/mm^−1^ | 2.104 | 1.976 |
| *F*_000_ | 676.0 | 676.0 |
| Reflections collected | 11599 | 4194 |
| Independent reflections | 3298 | 1395 |
| *R*(int) | 0.0290 | 0.0344 |
| Completeness | 99.6% | 97.9% |
| Data/restraints/parameters | 3298/0/136 | 1395/0/69 |
| Goodness-of-fit on *F*^2^ | 1.029 | 1.004 |
| *R_1_*^a^ [I > 2σ(I)] | 0.0222 | 0.0510 |
| *ωR_2_*^b^(all data) | 0.0466 | 0.0510 |
| ^a^ *R_1_* = Σ\|\|*F*_o_\|-\|*F*_c_\|\|/Σ\|*F*_o_\|. ^b^ *ωR_2_* = {Σ[ω(*F*_o_^2^-*F*_c_^2^)^2^]/Σ[ω(*F*_o_^2^)^2^]}^½^ | | |

**Supplementary References:**

1 Ichikawa, M. & Matsuo, T. Deuteration-induced structural phase transitions in some hydrogen-bonded crystals. *J. Mol. Struct.* **378**, 17-27 (1996).

2 Maczka, M. *et al.* Order-disorder transition and weak ferromagnetism in the perovskite metal formate frameworks of (CH_3_)_2_NH_2_ M(HCOO)_3_ and (CH_3_)_2_ ND_2_ M(HCOO)_3_ (M = Ni, Mn). *Inorg. Chem.* **53**, 457-467 (2014).

3 Horiuchi, S., Kumai, R. & Tokura, Y. Room-temperature ferroelectricity and gigantic dielectric susceptibility on a supramolecular architecture of phenazine and deuterated chloranilic acid. *J. Am. Chem. Soc.* **127**, 5010-5011 (2005).

4 Yao, Z.-S., Yamamoto, K., Cai, H.-L., Takahashi, K. & Sato, O. Above room temperature organic ferroelectrics: Diprotonated 1,4-diazabicyclo[2.2.2]octane shifts between two 2-chlorobenzoates. *J. Am. Chem. Soc.* **138**, 12005-12008 (2016).

5 Chen, K.-J. *et al.* Rapid and reversible photoinduced switching of a rotaxane crystal. *Nature Commun.* **7**, 13321 (2016).

6 Das, D., Jacobs, T. & Barbour, L. J. Exceptionally large positive and negative anisotropic thermal expansion of an organic crystalline material. *Nature Mater.* **9**, 36 (2009).

7 Spackman, M. A. & McKinnon, J. J. Fingerprinting intermolecular interactions in molecular crystals. *CrystEngComm* **4**, 378-392 (2002).

8 Aizu, K. Possible species of “ferroelastic” crystals and of simultaneously ferroelectric and ferroelastic crystals. *J. Phys. Soc. Jpn.* **27**, 387-396 (1969).

9 Goodwin, A. L. *et al.* Colossal positive and negative thermal expansion in the framework material Ag_3_[Co(CN)_6_]. *Science* **319**, 794 (2008).

10 Mary, T. A., Evans, J. S. O., Vogt, T. & Sleight, A. W. Negative thermal expansion from 0.3 to 1050 Kelvin in ZrW_2_O_8_. *Science* **272**, 90-92 (1996).

11 Cai, W. & Katrusiak, A. Giant negative linear compression positively coupled to massive thermal expansion in a metal–organic framework. *Nature Commun.* **5**, 4337 (2014).

12 Collings, I. E. *et al.* Homologous critical behavior in the molecular frameworks Zn(CN)_2_ and Cd(imidazolate)_2_. *J. Am. Chem. Soc.* **135**, 7610-7620 (2013).

13 Ogborn, J. M., Collings, I. E., Moggach, S. A., Thompson, A. L. & Goodwin, A. L. Supramolecular mechanics in a metal–organic framework. *Chem. Sci.* **3**, 3011-3017 (2012).

14 Grobler, I., Smith, V. J., Bhatt, P. M., Herbert, S. A. & Barbour, L. J. Tunable anisotropic thermal expansion of a porous zinc (II) metal–organic framework. *J. Am. Chem. Soc.* **135**, 6411-6414 (2013).

15 DeVries, L. D., Barron, P. M., Hurley, E. P., Hu, C. & Choe, W. “Nanoscale lattice fence” in a metal–organic framework: interplay between hinged topology and highly anisotropic thermal response. *J. Am. Chem. Soc.* **133**, 14848-14851 (2011).

16 Yang, C., Wang, X. & Omary, M. A. Crystallographic observation of dynamic gas adsorption sites and thermal expansion in a breathable fluorous metal–organic framework. *Angew. Chem. Int. Ed.* **48**, 2500-2505 (2009).

17 Wei, Y.-S. *et al.* Turning on the flexibility of isoreticular porous coordination frameworks for drastically tunable framework breathing and thermal expansion. *Chem. Sci.* **4**, 1539-1546 (2013).

18 Zhou, H.-L. *et al.* Direct visualization of a guest-triggered crystal deformation based on a flexible ultramicroporous framework. *Nature Commun.* **4**, 2534 (2013).

19 Zhou, H.-L., Zhang, Y.-B., Zhang, J.-P. & Chen, X.-M. Supramolecular-jack-like guest in ultramicroporous crystal for exceptional thermal expansion behaviour. *Nature Commun.* **6**, 6917 (2015).

20 Huang, Y.-G. *et al.* Superior thermoelasticity and shape-memory nanopores in a porous supramolecular organic framework. *Nature Commun.* **7**, 11564 (2016).

21 Pang, J. *et al.* Visualizing the dynamics of temperature- and solvent-responsive soft crystals. *Angew. Chem. Int. Ed.* **55**, 7478-7482 (2016).

22 Hutchins, K. M., Groeneman, R. H., Reinheimer, E. W., Swenson, D. C. & MacGillivray, L. R. Achieving dynamic behaviour and thermal expansion in the organic solid state via co-crystallization. *Chem. Sci.* **6**, 4717-4722 (2015).

23 Bauer, J. D. *et al.* Elastic properties, thermal expansion, and polymorphism of acetylsalicylic acid. *Cryst. Growth Des.* **10**, 3132-3140 (2010).

24 Panda, M. K. *et al.* Colossal positive and negative thermal expansion and thermosalient effect in a pentamorphic organometallic martensite. *Nature Commun.* **5**, 4811 (2014).

25 Fortes, A. D., Suard, E. & Knight, K. S. Negative linear compressibility and massive anisotropic thermal expansion in methanol monohydrate. *Science* **331**, 742-746 (2011).
