# Supplementary material for: Giant anisotropic thermal expansion actuated by thermodynamically assisted reorientation of imidazoliums in a single crystal
Source: Nat Commun. 2019 Oct 22;10:4805. doi: 10.1038/s41467-019-12833-y (PMC6805950; doi:10.1038/s41467-019-12833-y)
Supplement: Supplementary file 3 — Description of Additional Supplementary Files [file 41467_2019_12833_MOESM3_ESM.docx]

**Description of Additional Supplementary Files**

**File Name: Supplementary Movie 1**

**Description:** The thickness of single crystal expanded by 10% upon heating from 123 K to 403 K. The movie was constructed from the photographs recorded at 20 K intervals. The crystal was glued onto a glass fiber and enveloped in a temperature-controlled stream of dry nitrogen gas.

**File Name: Supplementary Movie 2**

**Description:** The molecular dynamic simulations at 123 K. The Himd+ cations demonstrate an in-plane wobbling at 123 K.

**File Name: Supplementary Movie 3**

**Description:** The molecular dynamic simulations at 413 K. The Himd+ cations become rotation around its molecular C5-axis upon heating to 413 K.
